# Semantics for Subjective Measures of Perceptual Experience

**DOI:** 10.3389/fpsyg.2019.01980

**Published:** 2019-08-27

**Authors:** Pessi Lyyra

**Affiliations:** Department of Psychology, University of Jyväskylä, Jyväskylä, Finland

**Keywords:** subjective measures of consciousness, introspective measures of consciousness, introspection, confidence rating, metacognition, experiential concepts

## Introduction

After decades devoted to appreciating the subjective character of consciousness, the discipline of consciousness research still seems haunted by how to measure subjective perceptual experience. In visual tasks, performance as an objective measure typically reflects the quality of perceptual experience. Close to the perceptual threshold, however, non-conscious perception begins to undermine the validity of this approach. Providing support for absence of conscious awareness of a stimulus, inferred from participant's inability to report on it (Hannula et al., 2005), is possible using statistical power, confidence intervals or Bayesian statistics (Dienes, 2014). However, objective performance-based measures of perceptual experience remain indirect and limited in scope.

Self-ratings of performance (confidence ratings/post decision wagering) could reflect perceptual experience, even though temperament and response style bias the data (Schurger and Sher, 2008). Recent studies indicating that confidence processing can work independently of perceptual experience further threaten the validity of these measures (Zehetleitner and Rausch, 2013; Jachs et al., 2015). To date, “introspective” measures, designed to capture conscious perceptual experience as “stimulus visibility” (e.g., Sergent and Dehaene, 2004; Rausch and Zehetleitner, 2014; Rausch et al., 2015), or by its clarity (Ramsøy and Overgaard, 2004; Overgaard et al., 2006) seem the most promising ones. Overgaard et al. (2006), however, criticize measures using “stimulus visibility” as ambiguous; the participant may be reporting on either the stimulus or the perceptual experience. The authors present their Perceptual Awareness Scale (PAS) as a less ambiguous alternative (p. 702):

“When using PAS, it has been considered imperative that subjects be clearly instructed that they are to report “introspectively” (Ramsøy and Overgaard, 2004). That is, they are to report about what they experience rather than report about stimulus features. […]”

Because of the “transparency of experience” (e.g., Tye, 2002)—what we perceptually experience seems nothing over and above the stimulus features—what does it exactly mean to report on experience instead of stimuli? Subjective scales typically offer no further instruction or explanation, and the working of introspection has been largely taken for granted. Some promising, more philosophical views on introspection have been suggested (e.g., Hatfield, 2005; Gallagher and Overgaard, 2006), but they have not really affected the content or the use of subjective scales.

Persuh (2017) recently contested the introspective measures claiming that, once quantified, the reports obtained using these scale are no more subjective than any other behavioral data. The ratings amount to nothing more than reports about perceptual discrimination between stimulus and background, similarly as with an objective measure. He concludes that:

“[S]ubjective measures do not assess the subjective character of our visual experience any better than objective measures. It follows that the “subjective” character of subjective measures is illusory and that subjective measures, like objective measures, estimate only performance on a discrimination task.”

Introspective measures thus continue to confuse researchers. So, how can a researcher instruct participants to report on their experience reliably and validly using these scales? If we take seriously the special epistemology of subjective experience, we should be able to construe the same for introspective measures. This, in my view, requires further clarification of what kind of content introspection can have in subjective measures of perceptual experience.

## Introspective Content for Subjective Measures of Perceptual Experience

The hot potato for semantics of experience has been whether the worldly object or the perceiver's internal constitution determine the content of a given perceptual experience. If only the worldly objects, recall the transitivity principle, whenever describing properties of experience, we necessarily describe properties of perceptual objects. Introspection would not be possible in any meaningful sense (Dretske, 1995, 1999). In a visual threshold experiment, one could only report on a stimulus, as is the case with objective measures. Things get more interesting in trials with such false positives, in which a similar perceptual experience arises in the absence as in the presence of a stimulus. Studies using objective measures typically neglect these trials, or use them, together with false alarms based on motor errors or response bias, to estimate “response conservativity,” as with d' as described by signal detection theory (e.g., Green and Swets, 1988). If the instruction is to report introspectively on experience, not the stimulus, then this is a mistake: the presence or absence of the stimulus should not make any difference. A subjective, mind-dependent content is required for introspective measures.

Obviously, both the stimulus and the perceiver's constitution play a role in the normal case, and these determine distinct dimensions of experiential content (cf. Chalmers, 2002, 2004): In addition to the accuracy conditions determined by the stimulus, perception carries information about the *manner* the stimulus *appears* to the observer (cf. Gallagher and Overgaard, 2006). The manner of appearance depends on the perceptual system, the state, and the expectations of the perceiving individual, along with contextual factors. The manner of appearing constitutes a possible content for introspection, as it can be construed more or less independently of the stimulus or its features.

Introspective measures, nevertheless, require specific instructions for the individual to access the correct dimension of experiential content. Cognitively, the introspective shift of focus on subjective experience requires use of second-order, “experiential” or “phenomenal” concepts (e.g., Carruthers, 2000; Alter and Walter, 2007), which enable an individual to learn to focus and report on his/her experience instead of the stimuli. In the phenomenological theory, the corresponding conceptual shift, by which the naïve inclination about the reality of perceptual objects is suspended, is called “bracketing” (see, Tufford and Newman, 2012). Consider watching The Moon, and resist your naïve realist inclination. What remains for observation is your perceptual experience. You may discover that your visual field appears detailed around the point of focus, but becomes increasingly blurred toward periphery. Press your eyeball, and you may visually experience two blurred moons. You could even have a pareidolic experience as of a moon having a face (e.g., Liu et al., 2014). These are not stimulus features but those of your experience. Judgments about them are introspective judgments with their own stimulus independent accuracy conditions. The subject needs to be consulted about this experiential content, and no objective description can replace it.

Note that the specificity of the instruction determines the content of introspective reports. A veridical and a non-veridical stimulus may be experienced similarly. Even if the perceptual content were determined by the perceiver's state and constitution in the non-veridical case—only introspectable content were present—there would not be qualitative difference between introspection and perception! Instructed to report on the stimulus features, you would be mistaken about them, but when instructed to report introspectively using experiential concepts, you would be correct about your experience. Contrary to Persuh (2017), the reports can be quantified into data either reflecting subjective introspective content or objective accuracy, depending on the instruction. The objectiveness of the quantified data does not change what they represent.

Confidence judgments typically pose no challenge to individuals; we routinely assess our performance for accuracy, and their instructions do not require use of experiential concepts. Confidence processing occupies a central role in some recent influential representationalist frameworks, such as the “predictive processing” framework (e.g., Friston, 2010; Clark, 2013; Hohwy, 2013). On this view, perception is mostly top down prediction of the incoming input, based on a generative representational model of the world. Only the signal about the discrepant input travels bottom-up, and is used to update the model. For optimal updating, the input accuracy needs to be evaluated at every level of sensory processing. Our representations are thus statistical, including second-order statistics about the predicted signal-to-noise ratio of the sensory input. Confidence assessment thus permeates perception. If so, we should be able to say at which level of processing confidence ratings and introspective judgments in experimental tasks take place. My immediate impression is that confidence judgments are based on access to an end result of implicit confidence processing (see Hohwy, 2013, p. ch. 9), not dependent on conscious perception. Contrary to (Overgaard and Sandberg, 2012) view, compared to confidence measures, introspective measures thus seem to operate at a higher level of access to conscious contents, using experiential concepts, the application of which requires more training and conceptual care.

## Operationalizing Introspective Content

The construct validity of an introspective measure depends on how well the use of experiential concepts is operationalized. Subjective measures are typically, and purposefully (Ramsøy and Overgaard, 2004), simple one-item scales, and the 4-points of the original version of PAS focus on the subconstruct of clarity of experience:

1. No experience. No impression of the stimulus is experienced. All answers are experienced as mere guessing.
2. Brief glimpse. A feeling that something was present, even though a content cannot be specified any further.
3. Almost clear experience. Feeling of having seen the stimulus, but being only somewhat sure about it.
4. Clear experience. Non-ambiguous experience of the stimulus.

The items of PAS are mostly formulated using experiential, second-order concepts of “glimpse,” “feeling,” and “experience,” although alluding to the presence of “the” stimulus. The scale mostly seems a subjective measure of (the clarity of) visual experience, but some ambiguity is preserved, including its name. PAS validated by correlating it to stimulus durations (Overgaard et al., 2006), and its metacognitive sensitivity evaluated by comparing it with confidence measures for which scale best predicts performance accuracy (see, e.g., Overgaard and Sandberg, 2012; Rausch et al., 2015). However, if the point is to focus on experience, this approach seems questionable; clarity of experience typically accompanies high confidence, but these two are dissociable. This could be another reason, in addition to differences in level of cognitive processing, scale criteria, and quantifying methods for introspective and confidence judgments, why comparing the scales has yielded conflicting results. Choice of the scale should be carefully considered against the specific purpose of the study (Rausch et al., 2015). Introspective measures work best in probing the features of experiences *per se*, and PAS-like scale captures well the gradualness of experience. The scope of PAS, however, becomes limited moving away from temporal visual threshold paradigms, and there is a need for corresponding systematic introspective scales for other experimental paradigms.

As for the issue of how well the participants are instructed to report introspectively, Overgaard et al. (2006) insist that participants can manage this by sufficient training (cf. Lutz and Thompson, 2003). Given this, (Persuh, 2017) claim that PAS is a measure of figure–background discrimination, rather than introspective measure of subjective perceptual experience, seems unfounded.

The visibility scales, in turn, seem to occupy an intermediate position between introspective and performance measures. “Stimulus visibility” implicates both the visual experience and the presence of the stimulus, unless more specifically instructed. The intentionality of experience is included in the judgments about stimulus visibility. They could thus qualify half-introspective, conforming to the idea of two-dimensional semantics of experience. Some subjective measures of interoceptive bodily perception, such as pain (Garra et al., 2010), or valence (Bradley and Lang, 1994) may similarly appear ambiguous between experiences and bodily states; the manner of how the body is perceived seems to be built into them. These measures may also qualify as partly introspective. This is not necessarily a setback for the introspective nature of these measures, as long as researchers keep the difference between the two dimensions of their content duly in mind.

## Relevance for Consciousness Research

The most important contribution of clarifying the semantics of introspection to consciousness research is providing conceptual resources for researchers to evaluate and develop their measures—how much they pertain to experience and how much to accuracy of perception about stimulus features. Keeping this in mind, the more ambiguous, or better “two-dimensional” scales work as proxies for introspection in the right context. The same should hold for objective behavioral or neural measures. A case can be made, though, that these measures acquire their validity from introspective reports, information about stimulus presence, and the divergence between them. Genuinely introspective reports are thus paramount for seeking the objective, neural or behavioral, correlates of perceptual experience, especially when we wish to correlate experience—not the presence of the stimulus—with our neural and behavioral responses.

## Author Contributions

The author confirms being the sole contributor of this work and has approved it for publication.

### Conflict of Interest Statement

The author declares that the research was conducted in the absence of any commercial or financial relationships that could be construed as a potential conflict of interest.
